# Primary Disease Prevention for Southwest American Indian Families During the COVID-19 Pandemic: Camp in a Box

**DOI:** 10.3389/fsoc.2021.611972

**Published:** 2021-03-09

**Authors:** Francine C. Gachupin, Elissa Caston, Christine Chavez, Jacob Bernal, Phoebe Cager, Drew Harris, Tara John, Joe Remitera, Charlotte A. Garcia, Victoria M. Romero, Karina E. Gchachu, Celeste R. Gchachu, Kutz Garcia, Vincent Gchachu, Brenna M. Gchachu, Evelyn Rens, Jacquanette Slowtalker, Robert Blew, Keyauni Tracy, Ty Figueroa, Cynthia A. Thomson, Noshene Ranjbar, Melanie Hingle, Teresia O’Connor, Denise J. Roe, Vernon Grant, Shayna Swick, Jennie R. Joe

**Affiliations:** ^1^ Department of Family and Community Medicine, College of Medicine, University of Arizona, Tucson, AZ, United States; ^2^ University of Arizona Cancer Center, Tucson, AZ, United States; ^3^ Scottsdale, AZ, United States; ^4^ Tucson, AZ, United States; ^5^ Rio Rancho, NM, United States; ^6^ Department of Nutritional Sciences, College of Agriculture and Life Sciences, University of Arizona, Tucson, AZ, United States; ^7^ Mel and Enid Zuckerman College of Public Health, Tucson, AZ, United States; ^8^ Department of Psychiatry, College of Medicine, University of Arizona, Tucson, AZ, United States; ^9^ USDA/ARS Children’s Nutrition Research Center, Baylor College of Medicine, Houston, TX, United States; ^10^ Center for American Indian and Rural Health Equity, Montana State University, Bozeman, MT, United States; ^11^ University of Arizona, Tucson, AZ, United States

**Keywords:** obesity, diabetes, health promotion, healthy lifestyle, youth, parenting

## Abstract

The goal of the American Indian Youth Wellness Camp in a Box was to engage, educate and empower families to improve their health and overall well-being during the COVID-19 pandemic. Camp in a Box was a 9-week program, inclusive of a 1-week intensive camp component followed by an 8-week booster component with content focused on nutrition, mental health and physical activity education. The Camp in a Box is a Tribal/Urban Indian-University partnership, and materials were developed to replace an existing weeklong residential camp and to comply with social distancing guidelines. Fourteen American Indian families from Tribal/Urban Indian communities in the southwestern United States participated (36 children aged 2–18 years; 32 adults). The intensive camp week included daily materials for families to complete together, Monday through Friday. Materials were provided for approximately 4 h of activities per day. The booster sessions began after camp week and included approximately 4 h of supplementary activities designed to be completed at any time most convenient for the family over the course of the week. Activities were designed to encourage interaction among family members with materials and supplies for parents and youth to participate. Self-reported outcomes suggested that families changed their eating habits to include more vegetables, less sweets and junk food. Parents reported an increase in family physical activity and that the activities brought the family closer together. Our Camp in a Box program was feasible and well-received until school began. During camp week, 100% of recruited families participated; at Booster Week 8, ten families (71%) remained enrolled and active. Camp in a Box is a feasible alternative to residential camps for promotion of health behaviors associated with metabolic disease prevention among American Indian families. In contrast to residential camps for youth, Camp in a Box offers an opportunity to engage the entire family in health promotion activities.

## Contributions to the Field

The COVID-19 pandemic has underscored American Indian health disparities and the importance of primary prevention of comorbid conditions, many of which stem from obesity. Obesity is highly prevalent among American Indian children and adolescents. Youth with obesity are at higher risk for type 2 diabetes, hypertension and dyslipidemia, non-alcoholic fatty liver disease, and breast or colorectal cancers. Obesity during childhood and adolescence is a strong predictor of adult obesity and there is a critical need to intervene. The American Indian Youth Wellness Camp in a Box was to engage, educate and empower families to improve their health and overall well-being during the COVID-19 pandemic. Self-reported outcomes suggested that families changed their eating habits to include more vegetables, less sweets and junk food. Parents reported an increase in family physical activity and that the activities brought the family closer together. Our Camp in a Box program was feasible and well-received. Camp in a Box is a feasible alternative to residential camps for promotion of health behaviors associated with metabolic disease prevention among American Indian families. “This program demonstrates self-sufficiency in American Indian families as they are able to learn, understand, and implement activities without direct supervision of program staff. This method is an approach that has potential to be replicated or modified with similar community-based programs and perhaps clinical approaches like a diabetes prevention program.”

## Introduction

The COVID-19 pandemic has underscored American Indian health disparities and the importance of primary prevention of comorbid conditions (Hatcher et al., 2020), many of which stem from obesity. Obesity is highly prevalent among American Indian children and adolescents (Story et al., 1999; Caballero et al., 2003; Gachupin et al., 2017), who suffer disproportionately from poverty, lack of basic infrastructure, historical trauma and poor access to resources. Youth with obesity are at higher risk for type 2 diabetes (Pinhas-Hamiel and Zeitler, 1996), hypertension and dyslipidemia (Freedman et al., 1999), non-alcoholic fatty liver disease (Cruz et al., 2005), and breast or colorectal cancers (Calle and Kaaks, 2004). Obesity during childhood and adolescence is a strong predictor of adult obesity (Spruijt-Metz, 2011) and there is a critical need to intervene. The American Indian (AI) Youth Wellness Camp program was developed to address this critical need, based on the concept that the health of American Indian youth is broader than the absence of disease and includes psycho-emotional stress management (Minich and Bland, 2013).

We previously developed a summer camp focused on promoting health and supporting American Indian youth and their families regarding healthy lifestyle changes, with an emphasis on physical activity, healthy eating, and mental health (Gachupin and Joe, 2017). The established residential camp program introduced and reinforced key health education topics for American Indian youth and communities. For example, the nutrition classes cover topics such as: introduction to traditional foods, how to read food labels, components of Choose MyPlate, and 8-5-2-1-0 messaging (8 h of sleep, 5 fruits and vegetables, 2 h of screen time, 1 h of physical activity, and 0 sugared beverages), with a particular focus on sugar-sweetened beverages. The physical activity sessions include traditional games, Zumba, yoga, circuit training, archery, basketball, kick ball, dodgeball, and hiking. With the COVID-19 pandemic, it was not appropriate to conduct the residential camp in its typical format; however, given the continued importance to continue to address obesity prevention among American Indian youth and their families, the remote, socially-distanced Camp in a Box was developed. Camp in a Box was conducted with the aid of camp materials sent through the U.S. Postal Service to participating families’ homes. We did not change the educational messaging component of our camp program, we changed the implementation approach to ensure engagement was conducted in a safe environment.

We utilized results from our previous work to inform our Camp in a Box focus and education messaging. For example, 2019 American Indian campers (aged 10–15 years) reported: 1) 15.2% sometimes do not have enough to eat; 2) 39.4% reported drinking more than one sugar sweetened beverage per day; 3) 42.5% reported eating deep fried foods such as French fries or potato chips more than once per day; 4) 81.8% reported being physically active for less than 60 min per day during the past 7 days; 5) 51.2% reported playing video or computer games on an average school day for three or more hours per day; 6) 45.5% reported watching TV for three or more hours on an average school day; and, 7) 35.5% reported being bullied on school property during the last 12 months (Gachupin et al., 2019a). Based on 24-h dietary recall data, American Indian youth diets were high in calories, fat, and sodium, and low in fiber, calcium, and potassium (Gachupin et al., 2019b). Very few youth met the recommendations set forth in the Dietary Guidelines for Americans 2015–2020 (U.S. Department of Health and Human Services and U.S. Department of Agriculture, 2015) for daily vegetable intake, and no participants met recommended limits on calories from solid fats and added sugars, which accounted for approximately 40% of total energy intake (primarily sugar sweetened beverages and snack foods).

Herein we describe how “camp” was continued and expanded to include families and adapted to function without face-to-face interaction with counselors. Our program did not charge camp fees or registration, adhered to the Centers for Disease Control and Prevention (CDC), Tribal/Urban Indian, State and City COVID-19 pandemic guidelines, and was offered distanced, as Camp in a Box.

## Materials and Methods

### Overview

The American Indian Youth Wellness Camp in a Box was a nine-week program made up of a one-week intensive camp with daily activities and eight weeks of booster materials and activities, all focused on healthy eating, mental health, physical activity education, and parenting support. The intensive camp week included daily materials and occurred Monday through Friday. Materials were provided for approximately 4 h of activities per day. The booster sessions began after camp week and included supplementary materials to continue healthy lifestyle education focused on nutrition, physical activity, mental health and parenting support. The booster activities could be completed at any time most convenient for the family over the course of the week and included materials for approximately 4 h of activity over the week. Activities were developed to encourage interaction among family members, for example, there were enough supplies provided within the box so that parents and youth had their own materials for participation. The instructions were written in large, user friendly font, easy to follow wording, and with descriptive images, so that steps were easy to follow. The materials for youth were written at the level of an adolescent reading and comprehension level (7th grade) and materials for parents were written at the level of a non-professional adult. Each set of instructions included a table of contents, step by step instructions, illustrated educational materials, and easy to follow work sheets targeted to the various topics covered in every session: nutrition, physical activity, mental health, and parenting support. To encourage family time, Camp in a Box included arts and crafts activities.

The Camp in a Box is a Tribal/Urban Indian-University partnership, and materials were developed to replace an existing weeklong residential camp and to comply with social distancing guidelines. The approach described within this article was developed in consultation with Tribal/Urban Indian partners. During camp planning meetings, the schedule of focus areas and topics to be covered for camp week and all eight booster sessions were developed. The Camp in a Box education content was largely adapted from the Centers for Disease Control and Prevention, and curricula used in other positive youth development camp programs (Gachupin and Joe, 2017; Gachupin et al., 2019c). Activities were designed to be interactive with attention to reading level and range of ages of family members. The program content operationalized Social Cognitive Theory (Badura, 2004) and the Information Motivation Behavioral Model (Fisher and Fisher, 2002) to address behavior change through the provision of relevant health information and, resources to promote skills and self-efficacy and thus advancement in motivation toward healthier behaviors and did so at the individual and family levels.

**FIGURE 1:**
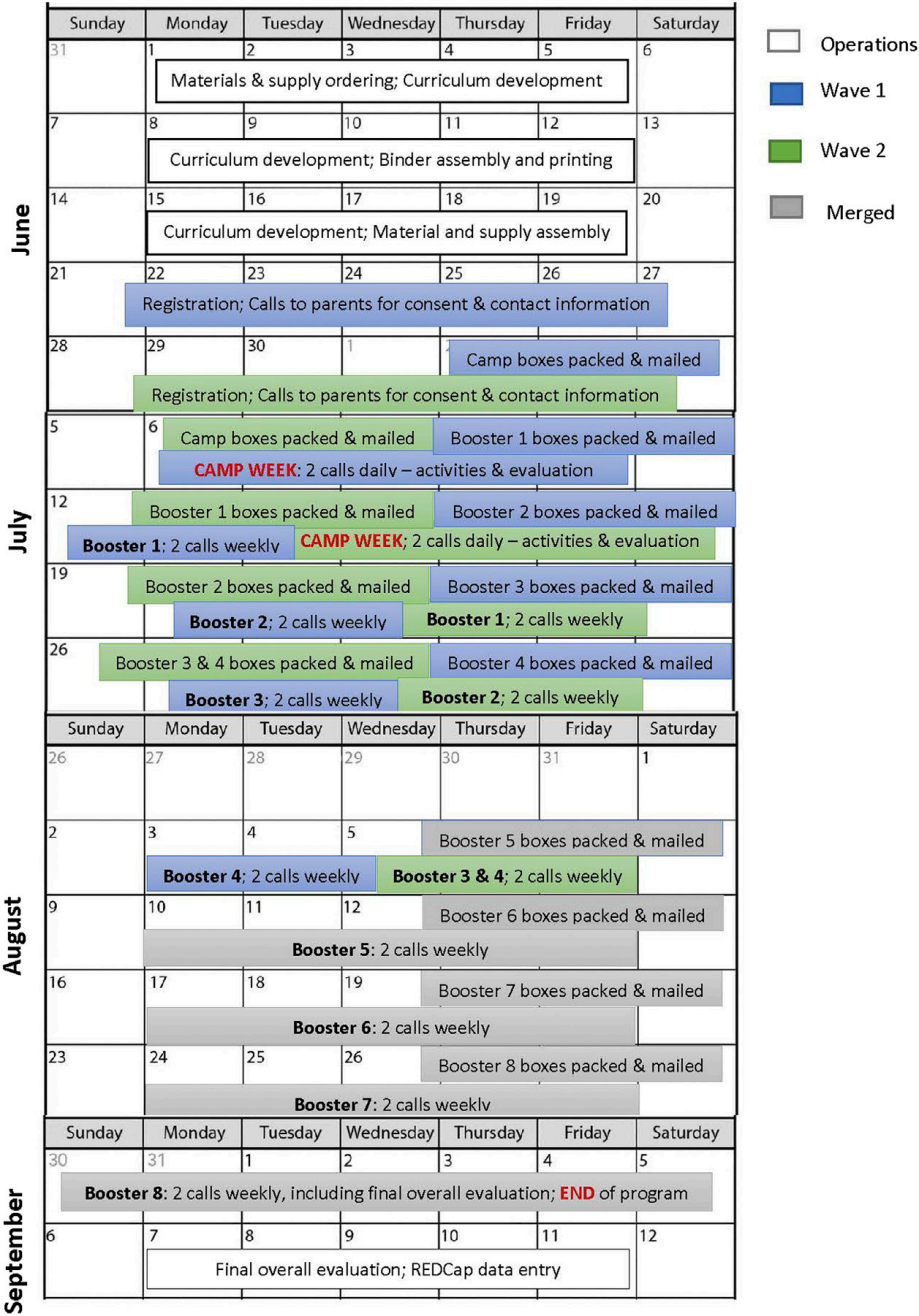
Southwest American Indian camp in a box program overview.

As individual specific activities were finalized, the supplies and materials needed were ordered. Although most likely related to the pandemic, many supply items had order limits placed on them. We often had to go through multiple vendors to fill the order for an item and depending on the demand of items, had to wait several weeks for delivery. All items were shipped to a Native woman owned shipping, receiving and fulfillment business where a 200 ft^2^ warehouse space was rented. Program materials for camp and booster activities were shipped in boxes on a weekly basis via the United States Postal Service to the participating families and Tribal/Urban Indian partners (see Figure 1). Activities were completed within private family residences on the families’ schedule. The camp, booster sessions, and all related communications were conducted in English. Camp in a Box occurred between July–September 2020.

### Camp Enrollment

Camp fliers were distributed to partnering Tribal/Urban Indian Health Department, Diabetes Program and Community Health or Wellness Program personnel who shared information with families within their respective Tribal/Urban Indian community or client lists. Interested American Indian families, who had at least one child between the ages of 10–15 years of age, were invited to participate. One parent from each interested family was asked to contact health program personnel to obtain registration materials which included the informed consent form, media release for photos, and the camp registration form. The registration form included demographic and household survey questions regarding family access to the internet, food security, and perceived stress. Program staff members verified consent and confirmed current and complete mailing address and cell phone number.

Families were enrolled in two waves, 11 families in Wave 1, and three families in Wave 2 (see Figure 1). The curricula and corresponding box materials were sent according to the family enrollment wave. Wave 2 began one week after Wave 1. A second wave was necessary due to additional families registering after the Wave 1 boxes had been mailed. Waves 1 and 2 were merged into a single group by booster session four (week five of Camp in a Box). This was achieved by consolidating two booster weeks into a single booster week for the Wave 2 participants, i.e., boxes containing instructions and supplies for both boosters were sent in the same week.

### Camp Staff Members

Camp in a Box program staff members included a camp director, two coordinators, two undergraduate students, and seven field-based team members (92% American Indian). Staff members participated in training led by the camp director prior to the beginning of camp and ongoing training as the camp curriculum unfolded. The trainings included chronic disease among American Indians; camp curriculum development; community-based participatory research; privacy and confidentiality; data collection, management and quality assurance; how to establish and maintain rapport; cultural sensitivity; COVID-19 impacts on Tribal/urban Indian communities; Tribal/Urban Indian partnership building; and university operational systems and policies. The coordinators and undergraduate students were responsible for designing camp activities, compiling camp education materials, obtaining consent, communicating with assigned families to confirm receipt of boxes and to complete oral evaluations. Evaluations were completed daily during the intensive camp week and once per week during the booster component. The coordinators and undergraduate students all worked remotely from their private residences for the entirety of Camp in a Box.

The field-based team members were responsible for reviewing camp and booster materials to prepare items and supplies needed for each box, which were personalized to families based on size of the family and sex and ages of the children. Camp in a Box items were mailed prior to the start of the camp day and booster week. The boxes included all the supplies needed for the various activities and were clearly labeled for each activity. Each box also included a healthy snack for each member of the family and no less than 30 boxes were shipped on a weekly basis. Preparations included addressing and labeling and database list maintenance as several families moved during Camp in a Box. An essential component of the initial box materials was a binder with activity instructions and reference materials, including education materials, for each day of the week. Materials for the booster sessions were sent weekly and included new instructions and educational content to be added to the binder. The binder was prepped with colored card stock dividers for addition of materials for subsequent booster sessions. All final materials were printed, collated, 3-hole punched and sorted into respective binders and folders on a weekly basis over the entirety of camp. The number of pages per session ranged from 31–47 pages. All activity related supplies, for example, arts and crafts, were combined in zip lock bags and clearly marked by activity so they were easy to locate for family members. A total of 470 Camp in a Box boxes were shipped or received from June–September 2020.

In addition, a non-Native medical student, under supervision of a board-certified child and adolescent psychiatrist trained in mind-body medicine, assisted with development of mental health education materials for Boosters 2 and 5–7. The material was adapted from The Center for Mind-Body Medicine’s standardized curriculum (Gordon et al., 2020). The same psychiatrist led six, 90-min evening mind-body skills groups; techniques taught included meditation, guided imagery, and the use of expressive writing, movement and drawings. These groups have been shown to improve mental health in youth and adults (Gordon et al., 2008; Staples et al., 2011; Gordon et al., 2016; Jones et al., 2020).

### Camp Curriculum (see Figure 2).

#### Nutrition

Nutrition education materials helped families to identify food groups using ChooseMyPlate (www.choosemyplate.gov) and My Native Plate (Indian Health Service, 2020), and to determine the sodium, saturated fat and sugar content of their foods through label reading. Other nutrition activities helped families understand energy balance and energy density (e.g., portion size, nutrient density), and provided guidance on selection of healthy snacks and the importance of growing vegetables. Because the pandemic has affected food security, income, and regular access to food sources (many tribes faced travel restrictions), nutrition educational materials also focused on menu planning and food purchasing on a fixed budget. Families also received healthy snacks (i.e. dried fruit, popcorn, rice crisps) each day of camp week and every week during booster sessions.

**FIGURE 2:**
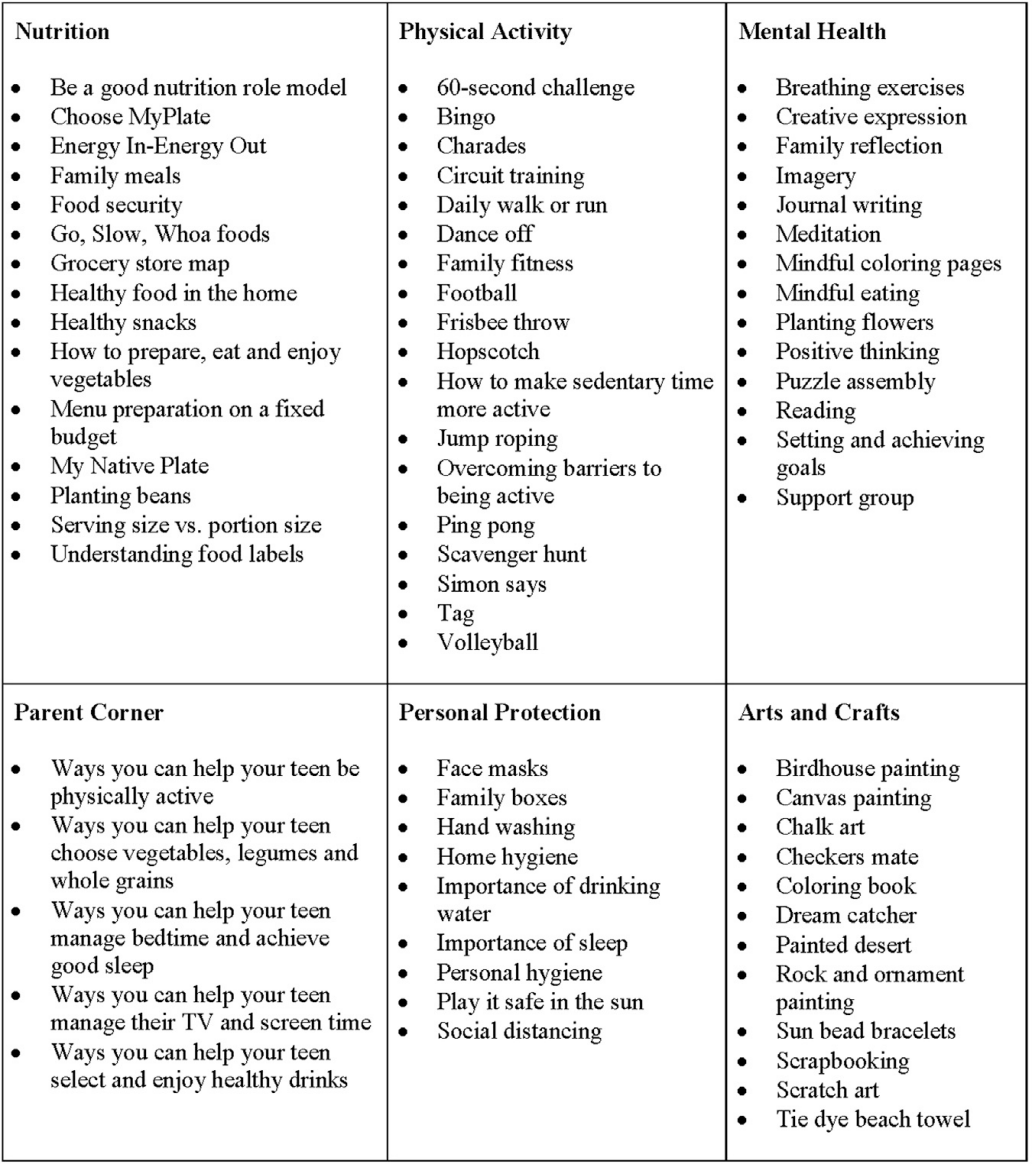
Southwest American Indian camp in a box program content.

#### Physical Activity

Physical activities included, for example, Frisbee throw games, circuit training, 60-s challenge, scavenger hunts, ping pong, charades, dance-off, hopscotch and a daily run or walk, either early or late in the day. Circuit training is comprised of individual exercise stations such as run in place, jumping jacks, lunges, frog jumps, crunches, squats, push-ups and wall sits, and participants are instructed to complete each exercise for 30 s at high intensity, modeled after the Indian Health Service Physical Activity Kit (U.S. Department of Health and Human Services, Indian Health Service, 2021). The participant then rotates to the next station and completes that exercise for 30 s at high intensity. The entire circuit is repeated for three rotations and participants are instructed to go at their pace and to drink water. The 60-s challenge was requested by a parent and participants were challenged to track how much of a given exercise can be completed in 60-s. The exercises included push-ups, lunges, jumping jacks, squats, sit-ups, tricep dips and tuck jumps (abdominal exercises). The participants were provided with sheets for daily and weekly tracking.

The necessary equipment was provided (i.e., Frisbees, ping pong paddles/balls, jump ropes) and written materials included detailed instructions, images of movements, work sheets, tracking sheets and links for additional resources. The physical activity materials were designed to support families in achieving the recommended 60 min per day of physical activity (U.S. Department of Health and Human Services, 2018; https://www.cdc.gov/physicalactivity/basics/index.htm). Activities and related materials were also designed to promote activity as an individual or family, to be adapted by a range of age groups and to reduce the risk for activity-related injury. When possible, physical activity promotion materials included alternative intensities and versions of specified activities, to allow multiple options for families to try. For example, for tag, instructions for freeze tag, hide and go seek tag, sock tag, triangle tag, and blob tag were included.

#### Mental Health

The mental health education materials included teaching the family about creative expression by providing them with conversation prompts and guidance for family self-reflections. Prompts were general and designed to foster journaling and family discussion. Examples of questions included: *A genie gives you three wishes, what are they?; You were granted a superpower today, what is it?; and, What’s a new trend you’d start tomorrow?* Additional mental health activities included breathing exercises, emotion expression, various forms of meditation, guided imagery, positive thinking, mindful eating, finding meaning and purpose, and reading. Some activities included the entire family, and some were specific for either just the youth (with parent guidance) or for the adults. The sessions covered self-expression through sharing in the group, as well as the use of drawings, music, movement, breathing techniques, guided imagery, body scan, and different forms of meditation. The mental health activities were created to be engaging and meaningful for American Indian youth and parents.

#### Parent Corner

Parenting education materials included strategies parents could use to help their children be successful in achieving health behavior goals of the program. Tips were based on food and physical activity parenting practices that promoted structure, responsiveness and autonomy support (Vaughn et al., 2016; O’Connor et al., 2017a; Mâsse et al., 2017). This included parenting practices that focused on creating a healthy home environment in which the parents served as role models and encouraged their child using positive language and clear expectations. Additionally, parents were encouraged to use autonomy supportive parenting practices to engage with their child in brainstorming activities to try, helping with grocery shopping, and preparing healthy dishes for the family. Parenting contexts covered physical activity, nutrition, screen media use and sleep, and corresponded to the other camp material content delivered that week. Additional parent focused education materials included how to prepare meals on a budget, how to make family time, how to talk back to negative thoughts, making healthy food more available and accessible in the home with work sheets, tracking sheets and links offered as additional resources.

#### Personal Hygiene

The personal hygiene education materials included COVID-19 prevention information (https://www.cdc.gov/coronavirus/2019-ncov/prevent-getting-sick/prevention.html), such as the importance of handwashing, use of face masks, social distancing, and sheltering in place, and other healthy living information, including sun safety (for example, use of sun screen, wearing long sleeves, limiting time in direct sunlight), importance of sleep, and proper hydration. Over the course of the nine weeks, several “family boxes” were delivered to the family residence, which included supplies to help the family achieve personal hygiene behavioral goals including use of sun screen, insect repellent, shampoo, hand soap, body soap, materials to make face masks, sun beads to monitor direct sun exposure, foot powder, dish soap, gloves, hand sanitizer, paper towels and napkins. The family boxes were in addition to the Camp in a Box materials.

#### Arts and Crafts

Each box contained arts and crafts materials to promote creative time including rock painting, making dreamcatchers, scratch art, tie-dye of beach towels, canvas painting, birdhouse painting and creating checker boards using duct tape. Supplies were provided so that *all* family members could participate, and all came with instructions to store the supplies for use in subsequent arts and crafts activities.

### Staff Follow-Up

Staff members were assigned individual households that were contacted once per day during camp week to review the daily activities and associated materials, and to ask the parent to evaluate the previous day’s activities. Each week of the booster sessions, calls were made by the same staff member early in the week to confirm receipt of the box(es) and at the end of the week to evaluate booster activities. Evaluation questions were specific to given activities. For example: *Did you review the food label materials?; Did your children write in their journal?; Will your kids play ping pong again?; and, Did your children enjoy planting beans?* The staff members scheduled the best day and time to call during the booster week with the respective parents for booster evaluations. The evaluation questions, directed to the parents, asked during booster weeks were general, open-ended questions. For example, *What did you like best about the booster activities this week?; What did you like least about the booster activities this week?; What did your kids like best about the booster activities this week?; What did your kids like least about the booster activities this week?; and, Do you have any suggestions?*

### Database and Institutional Review Board Approval

Staff members entered registration and evaluation responses in the REDCap electronic data management system at the University of Arizona, which provides secure storage and automated data export. Statistical analyses were performed using IBM SPSS Statistics, version 27.0 (Chicago, IL). The American Indian Youth Wellness Camp in a Box was approved by participating Tribal/Urban American Indian Organization, through their Tribal Leadership, Board of Directors and Tribal/Urban Indian Health Programs, and the university Institutional Review Board, 1911168177A001.

## Results

Demographics A total of 14 American Indian households (68 individuals; 100% American Indian) representing several different Tribal/Urban Indian entities in the southwestern United States participated. The households included 36 youth (aged 2–18 years; 62% female) and 32 adults including parents, grandparents, nieces, aunts, uncles, and cousins (aged 19–56 years; 79% female). The average number of children living in the house was 2.6 (range 1–6) and the average age of children living in the house was 11.7 years (range 2–18 years). Almost two-thirds (64%; *n* = 9) of families reported a family history of type 2 diabetes.

Fourteen adults completed the registration form to enroll their families in Camp in a Box. All parents completing the registration form were American Indian, a majority of whom were women (79%) (see Table 1). Most of our parents were employed and had more than a high school education. Over two-fifths (43%) of responding adults reported their health status to be either ‘excellent’ or ‘very good’. Over half of responding adults reported receiving public assistance. All responding adults reported having some sort of health insurance.

**TABLE 1:** Demographic characteristics, adult participants completing registration form (*n* = 14) variable.

	**Frequency (*n*)**	**Percentage (%)**
Sex		
Female	11	79
Male	3	21
Employment		
Full time work	11	79
Part time or temporary work	1	7
Unemployed (seeking work)	1	7
Unemployed (non seeking work)	1	7
Retired	0	0
Education		
Less than high school	1	7
High School/GED	4	29
More than high school	9	64
Health status		
Excellent	1	7
Very good	5	36
Good	4	28.5
Fair	4	28.5
Poor	0	0
Household benefits		
Food stamps	5	36
WIC	2	14
Unemployment	1	7
Social security	0	0
Veterans affairs	0	0
None	9	64
Health insurance		
Medicaid	7	50
Indian health service	4	29
Private insurance	2	14
Kidscare	1	7
Medicare	0	0
Uninsured	0	0

### Household Information

Over a quarter (28%) of responding adults reported their family was unable to get either food, clothing or utilities at some point in time during the past 12 months (see Table 2). Half of the responding adults reported being worried that food would run out often or sometimes, before getting money to buy more, during the past 12 months. Almost all (93%) of the participating families use their cell phone when accessing the internet. More than one-third of parents stated that internet access was costly, needed help on how to use the internet, or had very limited internet access, data not shown.

**TABLE 2:** Food security, internet access, and stress, adult respondents (*n* = 14).

**Question**	**Frequency (*n*)**	**Percentage (%)**
During the past 12 months family was unable to get		
Food	2	14
Clothing	1	7
Utilities	1	7
Other–Hygiene products	1	7
None	12	86
During the past 12 months, family worried that food would run out before getting money to buy more		
Often true	1	7
Sometimes true	6	43
Never true	7	50
Frequency see/talk to people you care about and feel close to		
Less than once a week	1	7
1 or 2 times per week	2	14
3 to 5 times a week	5	36
More than 5 times a week	6	43
Internet		
Home internet access	12	86
Use internet/Email	14	100
How do you access internet when you use it? (Choose all that apply)		
Cell phone	13	93
Tablet	7	50
Other—laptop 4; computer 3	7	50
How stressed are you?		
Not at all	2	14
A little bit	6	43
Somewhat	5	36
Quite a bit	1	7
Very much	0	0
Felt unable to control important things in your life		
Never	0	0
Almost never	0	0
Sometimes	3	21
Fairly often	6	43
Very often	5	36
Felt confident about ability to handle personal problems		
Never	3	21
Almost never	4	29
Sometimes	5	36
Fairly often	1	7
Very often	1	7
Felt difficulties were piled too high to overcome		
Never	1	7
Almost never	7	50
Sometimes	4	29
Fairly often	0	0
Very often	2	14

### Support, Stress and Anxiety

Although most Tribal/Urban Indian communities have strict social distancing rules and guidance, most responding adults reported being able to see or talk to people they care about and feel close to. Over three-quarters of responding adults reported feeling unable to control important things in their life, fairly or very often.

Beginning at Booster 4, weekly evening online mind-body skills groups were offered. The sessions ranged in attendance from 3 to 7 total participants, with at least one parent present at all sessions. A total of three different parents joined with one parent attending five sessions; two parents joined one session each.

### Overall Camp in a Box Evaluation

Overall evaluations of this pandemic-compliant program were highly favorable (see Tables 3 and 4). Parents reported that Camp in a Box instructions were easy to follow, informative, and that supplies were adequate to ensure participation by all. Parents reported that the Camp in a Box items were useful, and the family spent more time together than usual as a result of participating in Camp in a Box. The eight-week booster sessions inclusive of two calls per week from program staff to confirm receipt of boxes, review curricula, and evaluate the program were acceptable in frequency and duration. Parents reported that children were active for at least 60 min per day and they would now incorporate many of the lessons (e.g., healthy eating, physical activity, relaxation, arts and crafts) more regularly into the family schedule. Twenty percent of respondents said they would incorporate *all* of camp lessons now. A couple of parents also indicated they would continue to use the binder as reference.

**TABLE 3:** Camp in a box evaluation, questions following camp week.

**Question**	**Frequency (*n*)**	**Percentage (%)**
Were the instructions overall easy to follow?		
Yes	14	100.0
No	0	0.0
Were enough supplies provided for everyone to participate?		
Yes	14	100.0
No	0	0.0
Were your kids active for at least 60 min per day?		
Yes	14	100.0
No	0	0.0
Were the family box items provided useful?		
Yes	14	100.0
No	0	0.0
As a result of this week’s activities, did the family spend more time together than usual?		
Yes	11	78.6
No	3	21.4
*Camp in a box evaluation, questions following booster component*		
Were the two calls a week okay?		
Yes	12	92.3
No	1	7.7
Were the education materials informative?		
Yes	13	100.0
No	0	0.0
Were the 8 weeks of booster sessions okay?		
Yes	11	84.6
No	2	15.4
Is there anything from these lessons that you will now incorporate?		
Yes	13	100.0
No	0	0.0
If yes, please specify		
Nutrition (eating healthier, improving food habits)	6	25.0
Physical activity (dancing, jump rope)	4	16.7
All of them	4	20.0
Mind body medicine (relaxing, breathing techniques, being positive)	3	12.5
Arts and crafts (as a family)	2	8.3
Refer back to binder information	2	8.3
Increased family time/Communication	2	4.2
What did families like best about camp in a box? (Summed from all weeks’ responses; some named more than one thing in a given week)		
Arts and crafts (scrapbooking, scratch art, dream catcher, tie dye, checkboard, rock painting, chalk art, sunbeads)	55	36.2
All of it	21	13.8
Physical activity (60 s challenge, jump rope, circuit training, scavenger hunt, charades, frisbee, ping pong, walking)	19	12.5
Spending time with family	12	7.9
Snacks	11	7.2
Gave the kids something positive to do	7	4.6
Nutrition information (go slow whoa foods, vegetable bingo, planting beans)	7	4.6
Family box	6	3.9
Mind body medicine	5	3.3
Binder materials	2	1.3
Communication	2	1.3
Life lessons	2	1.3
Reading	2	1.3
Motivational messages	1	0.7
What did you families like least about camp in a box? (Summed from all weeks’ responses; some named more than one thing in a given week)		
Snacks	9	28.1
Journal writing	4	12.5
Online schooling makes it hard to do activities	4	12.5
Tie dye clean up	3	9.4
Checkerboard (duct tape stickiness)	3	9.4
Goal setting	2	6.3
Dreamcatcher (was frustrating)	1	3.1
Frisbee retrieval	1	3.1
Getting up early	1	3.1
Juggling work with camp	1	3.1
Mind body medicine	1	3.1
Morning walks	1	3.1
Take into consideration age gaps	1	3.1

**TABLE 4:** Camp in a box evaluation, questions following booster component.

**Question**	**Frequency (*n*)**	**Percentage (%)**
Were the two calls a week okay?		
Yes	12	92.3
No	1	7.7
Were the education materials informative?		
Yes	13	100.0
No	0	0.0
Were the 8 weeks of booster sessions okay?		
Yes	11	84.6
No	2	15.4
Is there anything from these lessons that you will now incorporate?		
Yes	13	100.0
No	0	0.0
If yes, please specify		
Nutrition (eating healthier, improving food habits)	6	25.0
Physical activity (dancing, jump rope)	4	16.7
All of them	4	20.0
Mind body medicine (relaxing, breathing techniques, being positive)	3	12.5
Arts and crafts (as a family)	2	8.3
Refer back to binder information	2	8.3
Increased family time/Communication	2	4.2
What did families like best about camp in a box? (Summed from all weeks’ responses; some named more than one thing in a given week)		
Arts and crafts (scrapbooking, scratch art, dream catcher, tie dye, checkboard, rock painting, chalk art, sun beads)	55	36.2
All of it	21	13.8
Physical activity (60 s challenge, jump rope, circuit training, scavenger hunt, charades, frisbee, ping pong, walking)	19	12.5
Spending time with family	12	7.9
Snacks	11	7.2
Gave the kids something positive to do	7	4.6
Nutrition information (go slow whoa foods, vegetable bingo, planting beans)	7	4.6
Family box	6	3.9
Mind body medicine	5	3.3
Binder materials	2	1.3
Communication	2	1.3
Life lessons	2	1.3
Reading	2	1.3
Motivational messages	1	0.7
What did you families like least about camp in a box? (Summed from all weeks’ responses; some named more than one thing in a given week)		
Snacks	9	28.1
Journal writing	4	12.5
Online schooling makes it hard to do activities	4	12.5
Tie dye clean up	3	9.4
Checkerboard (duct tape stickiness)	3	9.4
Goal setting	2	6.3
Dreamcatcher (was frustrating)	1	3.1
Frisbee retrieval	1	3.1
Getting up early	1	3.1
Juggling work with camp	1	3.1
Mind body medicine	1	3.1
Morning walks	1	3.1
Take into consideration age gaps	1	3.1

When asked what families liked best about camp, the overall program with the intensive camp week and booster sessions, 36.2% of responses related to the arts and crafts activities, including scrapbooking, scratch art, dreamcatcher, tie dye, checkerboard, rock painting, chalk art, and sun beads. Almost 14% of families liked all components of Camp in a Box. Twelve percent of respondents liked the different physical activities, such as the 60 s challenge, jump roping, circuit training, the scavenger hunts, charades, Frisbee, ping pong and walking. Families enjoyed having the chance to spend time together to do Camp in a Box activities, the snacks, and having their children doing something positive. The nutrition information and the family box, which contained household items, were liked by the families.

When asked what families liked least about camp, the overall program with the intensive camp week and booster sessions, the most frequent response (28.1%) was that they did not like specific snacks, such as apple chips, banana chips, dried apricots, and raisins. The popcorn received many positive comments and the beef jerky depended on variety, with different varieties liked better than others. Two parents liked the dry beef jerky so much that one asked for vendor information and another asked if it might be distributed again during Camp in a Box booster sessions. Additionally, twelve percent of respondents shared they did not like the journal writing activity and twelve percent responded it became hard to complete the booster activities once the school year started. Other respondents reported not enjoying the clean-up process following some of the camp and booster activities.

### Family Participation in Camp in a Box

Our Camp in a Box program was feasible and well-received until school began. We had 100% participation for camp week (see Table 5). With all students attending school online, the booster sessions quickly became burdensome. Although families requested to continue receiving weekly booster boxes, some could not complete activities within the prescribed week and could not complete evaluations. At Booster 8, we had retained ten families (71%). All but one family (who was lost to follow-up) completed the final overall camp evaluation (93%).

**TABLE 5:** Family participation in camp in a box.

**Camp Component**	**Number of Families Participating**	**Percentage (%)**
Camp week		
Monday	14	100
Tuesday	14	100
Wednesday	14	100
Thursday	14	100
Friday	14	100
Booster sessions		
Week 1	13	93
Week 2	14	100
Week 3	13	93
Week 4	12	86
Week 5	11	79
Week 6	11	79
Week 7	10	71
Week 8	10	71
Final overall evaluation		
Final survey	13	93

### Evaluation Communications

Over the course of Camp in a Box, there were 175 documented communications with parents (see Table 6). The most frequent ways the staff members communicated with the designated parent was through cell phone calls (97.1%). Most of the calls were with the mother and it often took a single call to get in touch (38.3%; 1–14 calls). Preferred time was established with the initial call and the calls occurred most frequently after work for calls that lasted between 5 and 10 min (45.7%).

**TABLE 6:** General contact information for camp and booster evaluations (*n* = 175).

**Variable**	**Frequency (*n*)**	**Percentage (%)**
Communication type for evaluation		
Cell phone voice	170	97.1
Cell phone text	4	2.3
E-mail	1	0.6
Communicated with		
Mother	130	74.3
Niece	21	12.0
Father	13	7.4
Grandmother	11	6.3
Number of contact attempts (Avg = 2.6)		
1	67	38.3
2	42	24.0
3	28	16.0
4	17	9.7
5	5	2.9
6	7	4.0
7	2	1.1
8	5	2.9
10	1	0.6
14	1	0.6
Time of call		
Before 10:00am	12	6.9
10:00am - 12:00pm	20	11.4
12:01pm - 5:00pm	68	38.9
After 5:00pm	75	42.9
Duration of call		
Less than 5 min	76	43.4
5–10 min	80	45.7
More than 10 min	18	10.3
Couldn’t connect	1	0.6

### Parent Comments

Parent communications were valuable and provided timely feedback. Several communications were especially heartwarming and some reinforced that the purpose of Camp in a Box was achieved:

*“I really enjoyed this camp. You guys have done an excellent job in providing educational value to us. I am very thankful for your camp because one of my kids was very closed off from the rest of us but because of this camp she broke out of her shell and demonstrated high levels of communication with us. Thank you, guys, again for this wonderful camp.”*

*“The activities really helped from the standpoint of bringing us closer together, it helped our communication outside of the camp.”*

*“Ideas from Booster 2 helped the entire family with meal planning.”*

*“We plan to do more meditation sessions and make it part of our weekly activities for the family. The kids are more aware of intensity of exercise now because of this booster.”*

*“We downloaded a weekly exercise journal from the CDC to keep track of exercises.”*

*“We liked the Rethink Your Drink activity because it’s a good reminder of what’s good/what’s not good and of all the sugar that can be in drinks.”*

*“We liked the Mindful Eating session. The kids usually don’t take their time with food and they eat really fast, so it was good to make them aware.”*

*‘It was exciting to learn about dream catchers and where they came from.”*

*“My daughter was looking forward to the tie dye project. She looked ahead in the schedule and was excited for Booster 6 art project.”*

*“I like all the information that you guys provide and how you guys walk us through step by step for the activities.”*

*“My daughter likes everything. She gets excited when things come in the mail.”*

### Social Media Metrics

Postings were made each day during the work week over the duration of camp to Facebook, https://www.facebook.com/AIWellness/. The posts included images of families completing the different camp activities, motivational messages, and reinforcement of education materials. Examples included reminders to exercise, to eat healthy meals, and to be mindful of stressors. Facebook metrics were collected from July 1–July 31 and from August 1–31. Camp in a Box had 190 followers in July with 186 Likes and a reach of 3,294 views. In August, there were 195 followers with 191 Likes and a reach of 2,392 views. There were 159 Shares in July and 119 Shares in August. Followers were primarily female (78%) aged 35–44 years of age (24%).

## Discussion

The key findings of our study are that a health promotion program with enhanced parental involvement can be implemented with relatively high response and retention rates during a pandemic (90.4% over entirety of Camp in a Box including final overall evaluation); that a health promotion program can be implemented without reliance on the internet; and, that written materials and minimal, yet essential and effective, communications with parents were sufficient to convey health promotion related information and elicit self-reported behavior change. These findings are especially important because the behavioral risks for chronic disease are high among American Indian youth and their families. It is exactly during times of challenge, such as the COVID-19, that prevention efforts focused on youth and their families need to be prioritized. Furthermore, the pandemic has placed heavy emphasis on digital learning, telehealth, Zoom meetings and our program provided valuable and needed reprieve from technology. “This program demonstrates the resiliency in American Indian families’ ability to learn in a non-virtual or in-person capacity.”

Findings presented herein contribute to our understanding of how to design, deliver and assess a health-themed, positive youth development (Catalano et al., 2004), family-oriented summer camp for American Indian children and adolescents. The goal of the American Indian Youth Wellness Camp in a Box was to engage, educate and empower families to improve their health and overall well-being. During pre-camp planning meetings with Tribal/Urban Indian-University partners, once it became clear that an in-person camp was not feasible because of the COVID-19 pandemic, our Tribal/Urban Indian partners shared their concerns about offering the camp via internet due to the expense, lack of access and/or lack of coverage on or near Tribal/Urban Indian lands. These internet issues were also mentioned by Press et al. (2003) and Wood et al. (2003). Through ensuing discussions with colleagues within the university, the “boxed” approach was suggested in adherence with CDC, Tribal/Urban Indian, state and city pandemic guidelines and recommendations. The proposed approach was shared with the participating Tribal/Urban Indian entities and once they were assured online links would be recommended as resources only, the Camp in a Box curriculum was developed.

The COVID-19 pandemic limited Tribal/Urban Indian engagement. Several Tribal/Urban Indian entities that were interested in participating in camp activities were not able to due to other community priorities and restrictions related to infection rates, shelter-in-place rules, and need to focus public health personnel on contact tracing and follow-up. In one participating community, recruitment had just begun when all Tribal/Urban Indian health personnel were mobilized for contact tracing and could no longer assist with other “non-essential” projects or programs. The Tribal/Urban Indian challenges of balancing pandemic response and continuing program deliverables were the reason we recruited families in two separate waves.

The Camp in a Box curriculum was designed to focus on education and resources to address the risk for obesity and related metabolic diseases such as cancer, cardiovascular disease and diabetes, and to be receptive to family experiences due to the COVID-19 pandemic. As evaluations were reviewed, the comments and suggestions were added to the existing curriculum. For example, we received requests for complex coloring sheets, puzzles, grocery store map, flower seeds and more challenging physical activities. In response, we incorporated circuit training and timed vigorous activities, such as 60 s challenge, as part of Boosters 3 and 7, sent more detailed coloring sheets as part of Booster 7, and included flower seeds and puzzles as part of Booster 8. Our overall curricula remains work in progress and we plan to complete additional formative qualitative work, for example, to adapt parenting materials to better meet the needs of American Indian families.

The entire Camp in a Box was designed to be user-friendly and to work within family constraints associated with the pandemic. We purposely limited communications to one designated adult per family for continuity of interactions and to cause as little stress as possible on families and communities alike. Based on questions included at registration, half of the parents (50%) worried that food would run out before getting money to buy more and 36% reported they felt unable to control important things in their life. Family boxes were sent in direct response to reported family food insecurity and included food sources. The finding that 86% of the participating households had home internet access did allow us to offer a weekly online mental health support group. Although the parent engagement with the mental health component was limited, those who did attend felt the sessions were extremely valuable.

Several of our findings may seem counter intuitive, for example, parents reported that their families liked arts and crafts the best and yet this camp is focused on addressing obesity. Southwest American Indians are well-known for their artistic talents and arts and crafts hold high importance and value within American Indian culture. There are research findings that Indigenous people who made traditional arts and crafts within the last year had reduced behavioral risks (Ryan et al., 2016). Furthermore, concepts related to American Indian “wellness include the creative arts, including art therapy, which have been beneficial in attending to spiritual and cultural values” (Herring, 1997).

Another finding that is somewhat contradictory is that select healthy snacks were not liked by some of the children and parents. It could be that these foods were unfamiliar to the family and that the new tastes of these foods were less than favorable. We did not ask if any of the provided snacks were new to the family, but several families volunteered information that some of the food items were indeed new to them. Our choice of healthy snacks was based on overall calorie count, sugar and salt content and price. Because our delivery was by postal mail, we could not ship fresh fruits and vegetables which would have been the ideal snacks to provide.

### Limitations

There are several limitations to mention. First, evaluations relied solely on parent self-report. Although we tried to accommodate parent availability, several parents felt bothered by our calls and requests to complete evaluations as demonstrated by quick, less thoughtful responses, and repeated rescheduling of calls. There were several instances where families fell behind on evaluations and had to be reminded which activities were being evaluated, so responses may have been influenced by accuracy of recall (Coughlin, 1990). Question content was developed to reflect the Camp in a Box objectives and to measure the impact of health promotion efforts on behavioral indicators such as knowledge, skills, motivation and self-efficacy. Although the research team did discuss the possibility of collecting minimal data, for example, weight, we did not pursue because we would not have been able to calibrate scales or verify information. We also did not want to introduce additional financial burden to families who may not have had weight scales.

Second, it is possible that Camp in a Box attracted families that were already more knowledgeable about or more invested in health practices than the average American Indian family of a similar demographic. It is not known how families who participated differed from those who did not participate. For the families that did participate, it worked well to have individual staff members assigned to work with given families for the duration of the program. The staff members were able to establish rapport to get to know the individual families and to understand and work with their unique situations and circumstances. To accommodate parent schedules, the staff members were extremely flexible and made calls in the evenings and sometimes on the weekends. To ensure our curriculum benefits all American Indian families, we have posted the curricula materials and activities on our website, https://www.fcm.arizona.edu/outreach/american-indian-youth-wellness-initiative/wellness-resources.

Third, a limitation of the intervention is its brevity. The United States Preventive Services Task Force guidelines for weight loss, behavior change, and cardiometabolic risk reduction in youth suggest high dose and long-term interventions (O’Connor et al., 2017b). Although Camp in a Box was nine weeks in duration, longer follow-up and more support and guidance to help participants sustain health behaviors would be ideal. Even with the nine-week intervention, the last few weeks were challenging for families. Our Camp in a Box program was feasible and well-received until school began. With students attending school online, the booster sessions quickly became burdensome for some families. It is unknown whether similar challenges would be encountered in a non-pandemic school year.

### Exploring New Directions Under the Impact of COVID-19

Across the United States, Tribal/Urban Indian entities have been disproportionately impacted by COVID-19 (Hatcher et al., 2020). Amid this devastating pandemic, it becomes even more important to engage American Indian families in primary prevention and health promotion initiatives to keep health disparities from increasing. The American Indian Youth Wellness Camp in a Box is an example of a program working to engage youth in healthy lifestyles, including mental health, and the inclusion of parents ensures a family-based approach and acknowledges Tribal/Urban Indian values. Parents can influence behaviors at home such as shopping for healthier foods and helping youth set limits on screen time. Evaluation results indicate that the program had a positive effect and increased participants’ knowledge, skills and behaviors regarding nutrition, physical activity, and mental health.

It is important to note that planning, staying on track, and organization were essential to Camp in a Box. Most of our weekly materials were not ready until the day before boxes needed to be shipped and much of the copying and collating that needed to be completed were within hours of the boxing of items to be shipped. The camp director and one field-based member were subject to strict tribal lock-down mandates for the entirety of Camp in a Box and had to fulfill program deliverables on designated travel days (Tuesdays and Saturdays) and return home before curfew. We know that parents were similarly constrained and some of our telephone communications were scheduled around local tribal curfews and COVID testing days.

Our program has the potential to make an impact on lifestyle choices in at-risk American Indian youth and thereby reduce the prevalence of youth at risk for obesity and related disease conditions such as diabetes and cardiovascular disease. Although there are select health promotion and disease prevention camps among American Indian communities, few assess impact on health (Teufel-Shone et al., 2009). Our plans for next steps are to explore hybrid approaches that continue some of the Camp in a Box core elements, to begin our intervention earlier in the summer so that conflicts with school are minimized, to expand our booster sessions for longer than two months and to expand our involvement of parents, and to utilize feedback received from program participants in all future planning, implementation and evaluation endeavors. Through our social media posts, we received several requests from American Indian families for more information on the camp, so we know there is interest in the curriculum and materials.

Now is the time to invest in promoting American Indian family resilience and enhancing physical and mental health and well-being in their respective communities and we are committed to answer the challenge.

## Data Availability

The original contributions presented in the study are included in the article/Supplementary Material, further inquiries can be directed to the corresponding author.
